# Stable incidence of surgical treatment and hospitalisation for humeral shaft fractures among 0- to 16-year-old patients in Finland from 1987 to 2010

**DOI:** 10.1007/s11832-014-0568-1

**Published:** 2014-02-20

**Authors:** A. Salonen, O. Pajulo, T. Lahdes-Vasama, V. M. Mattila

**Affiliations:** 1 Department of Pediatric and Adolescent Surgery, Pediatric Clinics and Pediatric Research Centre, Tampere University Hospital, Teiskontie 35, 33521 Tampere, Finland; 2 Department of Pediatric Surgery, Pediatric Clinics, Turku University Hospital, Turku, Finland; 3 Division of Orthopedics and Traumatology, Department of Trauma, Musculoskeletal Surgery and Rehabilitation, Tampere University Hospital, Tampere, Finland; 4 Department of Orthopedics, Karolinska Hospital, Stockholm, Sweden

**Keywords:** Adolescent, Humeral shaft fracture, Epidemiology, Incidence, Treatment

## Abstract

**Background:**

Studies among children experiencing fractures report an increasing trend toward operative management. In the present study, we examined whether the same trend has occurred for humeral shaft fractures in accordance with increasing interest toward intramedullary nailing and other operative treatments. The number, incidence and treatment of all hospitalised 0- to 16-year-old patients with humeral shaft fractures in Finland was assessed over a recent 24-year period.

**Method:**

The study included the entire adolescent (0–16 years) population in Finland during the 24-year period from January 1, 1987, to December 31, 2010. Data on hospitalised patients who sustained humeral shaft fractures were obtained from the nationwide National Hospital Discharge Register (NHDR) of Finland.

**Results:**

During the study period, there were a total of 1,165 hospitalisations with a main or secondary diagnosis of humeral shaft fracture. The incidence of hospitalisation due to humeral shaft fractures was 4.8 per 100,000 person-years. The incidence increased only slightly among girls from 3.3 per 100,000 person-years in 1987 to 5.3 per 100,000 person-years in 2010. The incidence of reposition and casting was 1.1 per 100,000 person-years and the incidence of reposition with osteosynthesis, including intramedullary nailing, was 1.4 per 100,000 person-years. The specific incidence of intramedullary nailing remained low with no signs of increased incidence, and the incidence was 0.3 per 100,000 person-years. There were no significant changes in the incidence of surgical treatment during the 24-year study period.

**Conclusion:**

Despite an overall increasing trend toward operative management of fractures in children, conservative management remains the treatment of choice for humeral shaft fractures based on the low and steady incidence of surgical treatment during the 24-year study period. In addition, the incidence of hospitalisation for fractures remained low without a significant increase during the study period.

## Introduction

Although humeral shaft fractures are relatively uncommon, they occur in every age group. Two peaks in occurrence are observed: in children under 3 years of age and in adolescents over 12 years of age [1, 2]. In general, humeral shaft fractures represent <10 % of humeral fractures in children and 1–3 % of all fractures in children [1, 2]. The most common fracture mechanism is direct trauma or rotational forces upon the humeral shaft. In newborn babies (birth weight over 4.5 kg), humeral fractures are considered to be due to birth trauma [3]. In children under 3 years of age, child abuse must be considered a potential cause of fracture [4]. In adolescents, most humeral shaft fractures are caused by sporting injuries [5].

The majority of humeral shaft fractures in children can be managed without surgery, although angulation may be difficult to control. The remodelling potential of the humerus is remarkable, and functional outcomes are still good, despite radiographic angulation [1]. A fractured humerus in children under 12 years of age can be remodelled with up to 70° of anterior angulation, and older children can tolerate anterior angulation of up to 30–40° in the upper arm [1]. The remodelling process cannot correct a malaligned rotational deformity, however, which, in severe cases, may lead to functional impairment in adolescents near adult age [6]. Surgical indications are controversial, but open fractures, bilateral fractures and fractures associated with multiple trauma, as well as arterial injuries, some nerve injuries and inadequate closed reduction, are considered indications for surgery [5]. Despite good results after conservative treatment, interest toward surgical stabilisation in adolescents with elastic titanium nails has increased [7, 8].

This study aimed to assess the incidence of surgery and hospitalisation for humeral shaft fractures among children 0–16 years of age in Finland. We also describe whether the trend toward surgical treatment changed during the study period, between 1987 and 2010.

## Materials, methods and statistical analysis

This study covered the entire paediatric and adolescent population (aged <17 years) of Finland during a 24-year period, from January 1, 1987, to December 31, 2010. Humeral shaft fracture data were obtained from the statutory, computer-based National Hospital Discharge Register (NHDR) of Finland. The Finnish NHDR was founded in 1967 and the information is collected equally from all hospital categories (private, public and other). The NHDR contains data on the age, sex and domicile of the subject; length of hospital stay; primary and secondary diagnosis; and operations performed during the hospital stay. The validity of the NHDR is excellent regarding both coverage and accuracy of the database [9–11].

The main outcome variable for this study was the number of surgically treated patients hospitalised with a main or secondary diagnosis of humeral shaft fracture (ICD-9 codes 8122A and 8123A in 1987–1996 and ICD-10 code S42.3 in 1997–2010). During the study period, the procedural codes changed. The procedure codes were ICD-9 from 1987 through 1996 and ICD-10 from 1997 through 2010. The ICD-9 procedural codes included in the study were 9123 (reposition and cast), 9126 (closed reposition and osteosynthesis) and 9128 (open reduction and osteosynthesis). The corresponding ICD-10 codes were NBJ41 (reposition and cast), and NBJ60 and NBJ40 (reposition and osteosynthesis).

For the purpose of analysing incidence trends during the study period from 1987 to 2010, the ICD-10 procedure codes were pooled with the ICD-9 codes. Treatment in the operating room was categorised into two groups; reposition with casting and reposition with osteosynthesis. Patients were analysed in three groups according to age: 0–6 years, 7–12 years and 13–16. Due to the small number of events in specific sex and age groups, operation-specific incidence rates were pooled for boys and girls.

To calculate the incidence of humeral shaft fractures leading to surgery and inpatient hospital treatment, the annual mid-population was obtained from the Official Statistics of Finland, an electronic national population register [12]. Statistical analysis was performed using PASW 19.0 (IBM SPSS, Chicago, IL, USA). The incidence figures were, thus, the true results concerning the entire adolescent population in Finland, rather than cohort-based estimates during the study period, and, therefore, 95 % confidence intervals were not calculated.

## Results

A total of 1,165 hospitalisations for patients from 0 to 16 years of age with a main or secondary diagnosis of humeral shaft fracture were registered during the 24-year study period. Boys comprised the majority of patients (62 %, *n* = 719). Surgical treatment was required in 585 (51 %) of the cases. The most common treatment method was repositioning and osteosynthesis (55 %, *n* = 323), including 79 fractures treated with intramedullary nailing (eight cases in those aged 0–6 years, 28 in those aged 7–12 years and 43 in those aged 13–16 years). Closed reposition and casting in surgery included 262 patients (45 %). Pain relief and further evaluation by senior paediatric orthopaedists was the reason for hospitalisation in 580 (49 %) of the cases in which no operations were performed. The mean age of the hospitalised children was 10.5 years (10.8 in boys and 10.1 in girls, *p* = 0.003).

During the study period, the incidence of surgery did not change. The incidence of repositioning and casting was 1.1 per 100,000 person-years during the 24-year study period (Table 1). The incidence of repositioning and casting was lowest in patients aged 13–16 years, with a mean of 0.9 per 100,000 person-years, and highest in patients aged 7–12 years, with a mean of 1.3 per 100,000 person-years. The incidence increased slightly in the youngest study group, those aged from 0 to 6 years, from 1.3 per 100,000 person-years between 1987 and 1997 to 1.7 per 100,000 person-years between 2000 and 2010. The corresponding incidence was 1.2 per 100,000 person-years between 1987 and 1997 to 0.6 per 100,000 person-years between 2000 and 2010 in the oldest study group.

**Table 1:** Incidence of repositioning with casting per 100,000 person-years among girls and boys aged 0–16 years between 1987 and 2010

	0–6 years	7–12 years	13–16 years
1987	1.6	0.3	0.8
1988	0.7	0.8	1.2
1989	1.6	1.5	0.8
1990	0.9	1	1.9
1991	0.7	0.8	1.9
1992	1.1	1.3	1.1
1993	1.8	2	0.4
1994	1.5	1.8	1.2
1995	1.9	2.3	0.8
1996	2	2.4	1.5
1997	0.7	0.8	1.1
1998	0.9	1	1.5
1999	1.9	2	0.8
2000	1	1	0.8
2001	1.5	1.5	0
2002	0.7	0.8	0.8
2003	1.8	1.8	1.1
2004	1.5	1.6	0.8
2005	1.8	1.9	1.5
2006	0.5	0.6	0.4
2007	1.5	1.7	1.1
2008	0.2	0.3	0.8
2009	1	1.1	0.4
2010	0	0	0

The incidence of repositioning and osteosynthesis was 1.4 per 100,000 person-years (Table 2). The incidence was lowest in those aged 0–6 years, with a mean of 0.5 per 100,000 person-years, and highest in those aged 13–16 years, with a mean of 2.5 per 100,000 person-years. The incidence of repositioning and osteosynthesis increased slightly only in the oldest study group from 2.1 per 100,000 person-years between 1987 and 1997 to 2.6 per 100,000 person-years between 2000 and 2010. The total number of fractures treated with intramedullary nailing was 79. The incidence of intramedullary nailing was 0.3 per 100,000 person-years. The incidence was highest in patients aged 13–16 years (*n* = 43), with a mean of 0.7 per 100,000 person-years. The highest incidence, 1.4 per 100,000 person-years, occurred in 1997 in those aged 13–16 years, and after 1997, the incidence decreased to 0.6 per 100,000 person-years without any signs of an increase.

**Table 2:** Incidence of reposition with osteosynthesis per 100,000 person-years among girls and boys aged 10–16 years between 1987 and 2010

	0–6 years	7–12 years	13–16 years
1987	0	0.8	2.5
1988	0.2	0.8	1.2
1989	0.2	1.8	2.4
1990	1.1	0.8	0.8
1991	0.2	1	2.7
1992	0	0.5	0.8
1993	0.4	0.8	1.9
1994	0.4	0.5	1.2
1995	0.7	1.6	3.1
1996	0.9	1.6	1.9
1997	1.1	1.3	3.8
1998	0.9	1	3.4
1999	0.5	0.8	1.9
2000	0.5	2	2.4
2001	0.2	1.5	2.8
2002	0.2	1.5	2.4
2003	0.8	1.3	2.7
2004	0.5	1.3	5
2005	0.5	2.4	3.4
2006	0.2	1.9	1.5
2007	0.5	1.1	2.6
2008	0.5	0.6	3.4
2009	0.7	0.9	2.7
2010	0.7	2.6	2.4

In the present study, the person-based incidence due to the hospitalisation of humeral shaft fractures was 4.8 per 100,000 person-years (6.0 per 100,000 person-years in boys and 3.7 per 100,000 person-years in girls). The incidence increased among girls, from 3.3 per 100,000 person-years in 1987 to 5.3 per 100,000 person-years in 2010 (Table 3). In boys, the incidence of humeral shaft fractures decreased slightly from 6.7 per 100,000 person-years in 1987 to 5.9 per 100,000 person-years in 2010 (Table 4). The highest incidence of fractures was 9.6 per 100,000 person-years in boys aged 13–16 years. The lowest fracture incidence was observed in girls aged 0–6 years (2.3 per 100,000 person-years).

**Table 3:** Incidence of humeral shaft fractures per 100,000 person-years among girls aged 0–16-years from 1987 to 2010

	0–6 years	7–12 years	13–16 years
1987	1.3	5.2	3.4
1988	2.3	4.7	3.4
1989	2.3	6.3	5.7
1990	1.4	5.2	3.9
1991	2.8	5.2	3.1
1992	1.8	3.6	1.6
1993	2.3	6.8	3.9
1994	2.2	2.1	3.2
1995	2.7	6.4	3.9
1996	3.6	7.5	7.7
1997	4.6	3.4	3.1
1998	1.9	6.9	3.1
1999	2.4	3.2	3.9
2000	3.4	7.3	8.9
2001	1.5	4.7	5.7
2002	1	2	4
2003	1.5	4.2	3.9
2004	3.1	2.7	3.1
2005	1.5	3.8	5.4
2006	1	3.3	5.4
2007	1.5	2.3	4.6
2008	1.5	2.3	3.9
2009	1.5	1.8	4.7
2010	4.9	5.9	4.8

**Table 4:** Incidence of humeral shaft fractures per 100,000 person-years among boys aged 0–16 years from 1987 to 2010

	0–6 years	7–12 years	13–16 years
1987	2.6	5	12.3
1988	6.2	1.5	8.8
1989	2.2	5.5	7
1990	3.6	3.5	7.5
1991	2.7	6.5	12.6
1992	1.8	5.1	7.5
1993	2.2	4.5	6.8
1994	6	5	8.3
1995	4.3	5.1	8.2
1996	4.3	6.2	11
1997	4.4	6.7	19
1998	0.9	4.6	11.7
1999	1.8	6.5	13.5
2000	2.8	3	6.9
2001	2.3	4.5	3.9
2002	0.9	6.5	5.4
2003	4.4	6.1	8.3
2004	2.5	9.2	14.2
2005	3.9	6.3	14
2006	1.9	6.4	5.1
2007	4.8	9.3	12.6
2008	1.9	2.2	7.4
2009	0.9	5.6	9.1
2010	3.2	6.2	8.5

The mean duration of hospital stay for the entire study group was 2.6 days. The mean duration of hospital stay was 2.5 days for patients with reposition and casting, and 3.4 days for patients with reposition or reduction and osteosynthesis.

## Discussion and conclusions

The principal aim of the present study was to describe the incidence and trends of operative treatment for humeral shaft fractures among children and adolescents aged 0–16 years in Finland between 1987 and 2010. The main finding was that, despite the overall increase in surgical treatment in children and adolescents, the incidence of surgery for humeral shaft fractures remained low during the 24-year study period. Also, the incidence of humeral shaft fractures leading to hospitalisation remained low, with no significant changes during the study period.

Based on the previous literature, approximately one-third of children sustain at least one fracture before 17 years of age and the majority of the fractures occur in the upper limbs [13–15]. Antebrachium fractures represent 35 %, while humeral diaphyseal fractures represent less than 1 % of all fractures [13–17]. According to Mäyränpää et al. [18], the incidence of all fractures other than upper-extremity fractures has decreased significantly over the past two decades. Helenius and coworkers [19] recently reported that the incidence of hospital-treated upper-extremity fractures has increased by 23 % in Finland during the preceding 10 years. Based on our earlier study (Salonen et al. [20]) and the present study, it seems that the main reason for the increased incidence of hospital-treated upper extremity fractures is distal humeral fractures.

The incidence of surgery remained low and steady during our study period. Roughly half of the patients were treated surgically by repositioning and casting or by osteosynthesis. Despite the increasing interest toward intramedullary nailing, its role in the management of humeral shaft fractures has remained low in Finland. The highest incidence of intramedullary nailing was 1.4 per 100,000 person-years and, interestingly, it did not increase during the study period, although elastic medullary nailing was recently suggested to be a good alternative to conservative treatment [7, 8]. Fernandez et al. [8] reported 31 children with traumatic humeral shaft fractures treated with elastic stable intramedullary nailing. In their sample, five complications occurred, all concerning the indication for surgery or technical error (skin irritation, damage of the radial nerve etc.) [8]. All patients and parents were satisfied with the treatment and all children were able to return to their sporting activities after treatment [8]. Zatti et al. [21] reported 40 patients, 14 treated with elastic stable intramedullary nailing and 16 treated with AO plates. Both groups had the same fracture healing time and functional recovery, allowing for early motion. The surgical technique of elastic nailing is simple, safe and rather atraumatic, and, therefore, valid for routine use [21]. Gordon and Garg as well as Slongo described indications and techniques for flexible titanium intramedullary nailing. They both reported optimal results of fracture treatment provided the indication is valid and the appropriate technique is used [22, 23]. Although our study did not compare the results of surgical and non-surgical treatment, conservative treatment was most often used and there was no significant trend toward an increase in surgical treatment.

The previously reported overall increasing incidence of fractures may by due to changes in children’s activity patterns over time. In addition, new leisure-time physical activities, such as jumping on a trampoline, may increase the fracture incidence [24]. Hurson et al. [24] reported a dramatic increase in fractures and other trampoline-related injuries in Ireland. A similar trend was reported in the United States during the past 10–15 years [15].

In the present study, the incidence of hospitalised humeral shaft fractures was 4.8 per 100,000 person-years. To our knowledge, this is the first nationwide study to assess the incidence of hospitalisation due to humeral shaft fractures in children and adolescents. In our study, we observed a slight increase in hospitalisation due to humeral shaft fractures among girls. It must be considered, however, that humeral shaft fractures are relatively uncommon and the observed increase may have been due to annual normal variation. The low and relatively stable incidence of humeral shaft fractures can be accounted for by the injury mechanism. Shaft fractures require a rather uncommon trauma mechanism with twisting or transverse high-energy injury, which is often associated with multiple traumas [25–27].

The mean age at injury onset was 10.1 years, and the peak incidence occurred somewhat earlier in girls than in boys. The majority of patients were boys. These results correspond to those in previous reports [14]. The younger age of girls may be explained by differences in the pubertal growth of girls and boys. During the pubertal growth spurt, there is a relative decrease in bone mineral density due to bone expansion and insufficient mineralisation [28]. The greater frequency of fractures in boys, on the other hand, can be explained by differences in exposure time and in the intensity of their leisure-time sporting activities. In addition, some humeral fractures might be due to violence, which is more common among boys [29]. Boys’ violent actions are connected to leisure-time activities as well as to alcohol, and increase with age [29].

A strength of this study is the Finnish NHDR, which provides an excellent database of patients treated in hospitals during the last 24 years. In addition, treatment is equally available for all Finnish citizens and, thus. patients can be followed in the hospital discharge register by their personal identification number. The limitations of this study include the lack of separation between intramedullary nailing and plating during the time when the ICD-10 classification was used. Based on our analysis of ICD-9 coding, however, plating is rarely performed in children and adolescents. Further, the incidence reported in the present study is based on hospitalisation data on severe and unstable fractures. There may have been some patients treated as outpatients that are not included in this study.

To summarise, while the overall incidence of adolescent fractures has increased rapidly, the incidence of humeral shaft fractures has not changed markedly over the past 24 years. The incidence of surgical treatment has also remained steady, despite alternative treatment choices (e.g. elastic intramedullary nailing and plating).
